# Direct estimation of the spontaneous mutation rate by short‐term mutation accumulation lines in *Chironomus riparius*

**DOI:** 10.1002/evl3.8

**Published:** 2017-05-11

**Authors:** Ann‐Marie Oppold, Markus Pfenninger

**Affiliations:** ^1^ Senckenberg Biodiversity and Climate Research Centre Molecular Ecology Group 60325 Frankfurt am Main Germany; ^2^ Faculty of Biological Science, Institute for Ecology, Evolution and Diversity Goethe University 60438 Frankfurt am Main Germany

**Keywords:** Diptera, genomic base composition, insect, mutational spectrum bias

## Abstract

Mutations are the ultimate basis of evolution, yet their occurrence rate is known only for few species. We directly estimated the spontaneous mutation rate and the mutational spectrum in the nonbiting midge *C. riparius* with a new approach. Individuals from ten mutation accumulation lines over five generations were deep genome sequenced to count de novo mutations that were not present in a pool of F1 individuals, representing parental genotypes. We identified 51 new single site mutations of which 25 were insertions or deletions and 26 single nucleotide mutations. This shift in the mutational spectrum compared to other organisms was explained by the high A/T content of the species. We estimated a haploid mutation rate of 2.1 × 10^−9^ (95% confidence interval: 1.4 × 10^−9^ – 3.1 × 10^‐9^) that is in the range of recent estimates for other insects and supports the drift barrier hypothesis. We show that accurate mutation rate estimation from a high number of observed mutations is feasible with moderate effort even for nonmodel species.

**Impact Statement**

Changes in the genetic code, called mutations, are the basis and the fuel for the evolution of any organism, because only they provide the variation that is necessary for selection to act upon. We estimated the rate at which new mutations occur from one generation to another in the entire genome of an insect species, the harlequin fly *Chironomus riparius*. The chance of a mutation to hit a particular base in the genome during a generation is very rare (even rarer than being killed by a lighting strike) and it is therefore difficult to measure them. For that reason, only few direct mutation rate estimates are currently available, even though our understanding of the mutation process is fundamental to our understanding of evolution. Our study design combines two common methods, one of which is very costly and the other very time‐intensive, to a more efficient approach that can be applied to many organisms. We compared the mutation rate of *C. riparius* to available mutation rates from other insects (fruit fly, a butterfly, bee, and bumble bee) and found that these insects mutate at a similar rate, although their rates seem to depend on the number of reproducing individuals within a population. The *C. riparius* genome contains much more A and T than C and G bases. A comparison to fruit flies with more balanced genomes revealed more mutations where a base was either inserted or removed than changed. This suggested that the kind of mutations occurring depends on the nucleotide composition of the respective genome.

## Introduction

Being the ultimate source of genetic variation for evolution to act upon, mutation is certainly among the most important evolutionary processes. The per generation rate at which spontaneous mutations occur in the genome is the central parameter to estimate the effective population size on recent time scales (Charlesworth 2009) or in the course of population history (Schiffels and Durbin 2014), equilibrium of genomic base composition (Hiroshi Akashi and Eyre‐Walker 2012) and divergence times (Ho 2014). Yet, the spontaneous mutation rate (μ) is so difficult to measure directly that it has been rarely estimated up to now. Consequently, only very few eukaryotic direct μ estimates are currently available (Denver *et al*. 2009; Keightley *et al*. 2009; Ossowski *et al*. 2010; Keightley *et al*. 2014a; Keightley *et al*. 2015; Yang *et al*. 2015; Keith *et al*. 2016; Liu *et al*. 2016), scarcely representing biodiversity.

According to the drift‐barrier hypothesis, mutations are deleterious in most cases and therefore a selection pressure exists to suppress them. However, realized mutation rates are the result of random genetic drift setting a barrier to the effectiveness of selection improving the fidelity of replication. Thus, mutation rates should scale with effective population sizes (Lynch *et al*. 2016). Furthermore, there is a documented discrepancy between the many orders of magnitude of variation in population size and the much narrower distribution of diversity levels, originating from spontaneous mutations (Lewontin's paradox, Ellegren and Galtier 2016). More mutation rate estimates, would therefore be highly desirable to shed light on the evolution of μ and its associated ecological and evolutionary circumstances (Lynch 2011). The understanding of μ variation among insects is not yet fully resolved (Liu *et al*. 2016), and thus, another direct rate estimate of a nonsocial insect species will help to increase resolution.

Currently, two approaches are applied to directly estimate μ: mutation‐accumulation (MA) lines experiments and parent‐offspring trios (Keightley *et al*. 2009; Keightley *et al*. 2014a). In the MA lines approach, inbred lines are established and bred over many generations (Mukai and Cockerham 1977). Due to the almost absent effect of selection, all except of the most deleterious mutations become eventually fixed and are thus readily identified and confirmed (Denver *et al*. 2009). However, establishing inbred lines is not possible for all organisms, often require complex logistics to transfer generations, intensive care over long‐time spans and recessively deleterious mutations will be lost with their respective MA‐line. In addition, mutator alleles may become fixed, altering the estimated mutation rate (Haag‐Liautard *et al*. 2007). In the trio approach, parents together with their offspring are full genome sequenced (Roach *et al*. 2010). This has the advantage that the observed mutational spectrum includes also recessively deleterious mutations as they appear heterozygously in the offspring. Limitations arise from the large number of offspring needed to be screened for an appreciable number of mutations and the requirement to know the parents (Keightley *et al*. 2014a), which is difficult in some species.

In our estimation of μ in the nonbiting midge *Chironomus riparius*, we drew on the advantages of both approaches. We established ten MA‐lines over five generations and deep sequenced the genomes of a single individual per MA‐line. Because individual parenthood is difficult to determine in the swarm breeding *C. riparius*, we compared these individuals with the pooled full‐sibling offspring from the single egg‐clutch the MA‐lines were established from. This yielded an appreciable number of mutations, allowing an accurate estimation of μ.

## Methods

We used a strain of *C. riparius* that was established from a field population in Western Germany and kept since several decades in various laboratories for genetic and ecotoxicological research (“Laufer population”), from which also the *C. riparius* reference draft genome was sequenced (Oppold et al. 2017). Larvae from a single egg‐clutch were raised at 20°C under most permissive conditions concerning space and food (OECD 2004) to avoid selection. The offspring (F_1_) was allowed to reproduce. After successful reproduction, the adults of this first generation were collected to produce the reference pool (see below). Twenty of the clutches were used to establish as many mutation accumulation lines, ten of them as back‐ups for MA lines going extinct. These lines were reared as described above for additional four generations, always bringing only a single egg‐clutch into the next generation. In the fifth generation, a single individual from each of ten randomly chosen MA lines was retained for genome sequencing (Fig. S1). Siblings of each sequenced individual were kept for experimental mutation confirmation.

Due to swarm fertilization of females, it is not possible to unequivocally determine the parents of a particular egg‐clutch. To obtain a baseline against which to identify DNMs, we pooled 190 individual head capsules of their F_1_ offspring and sequenced their pooled DNA to an expected mean coverage of 60X as 150 bp paired‐end library on a Illumina HiSeq2500 platform, because the allelic composition of the F_0_ parents should be mirrored in the allele frequency of their offspring.

One female individual of each of the ten MA‐line was whole‐genome sequenced to an expected mean coverage of 25X on an Illumina HiSeq4000 platform. Library preparation of single individuals was performed with the KAPA HyperPrep Kit (KR0961, KAPA Biosystems) to yield enough DNA. The 150bp paired‐end reads were individually adapter clipped and quality trimmed, using Trimmomatic (Bolger *et al*. 2014). The cleaned reads of MA line individuals and the reference pool were then processed with the GATK‐pipeline (McKenna *et al*. 2010), that is mapped with bwa *mem* (v0.7.10–r789, Li and Durbin 2009) against the reference genome draft (NCBI accession number to be provided), duplicates marked with Picard (v1.119 available at http://picard.sourceforge.net), realignment around indels (insertion and deletions) and recalibration of bases with GATK. The raw reads and bam‐files for all individuals and the F1 pool can be found at ENA (Project number PRJEB18039).

We established a multistep pipeline in line with Keightley et al. (2014a) to identify potential DNMs and minimize false positives. Initially, we applied variant calling with the GATK UnifiedGenotyper (DePristo *et al*. 2011) individually for each MA‐line. The reference pool, reflecting the joint genotype of two diploid parent individuals, was treated alike with the exception that variant calling parameters were set as for a tetraploid individual (see above). We then intersected all ten resulting vcf‐files amongst themselves and with the reference pool simultaneously, retaining the unique variants for each MA‐line, using bcftool *isec* (v1.3, htslib 1.3, available at https://github.com/samtools/BCFtools).

Raw mutation candidates were then quality filtered following the GATK best practices (McKenna *et al*. 2010). Only candidate positions with an overall quality score (GQ) concerning base calling quality and position in read above 90 were considered. Variants with indication for substantial strand bias (SOR > 4) were removed. Since we wanted to concentrate on point mutations and single base indels, indel length was restricted to two. To assure sufficient coverage on the one hand and minimize the effects of mismapped duplicated, paralogous regions on the other, coverage depth was restricted to a range between 15 and 44 reads. A minimum allele count of five was required for the nonreference allele to be retained.

The resulting list of candidate positions was then used to create pileups between the respective individual and the reference pool using samtools *mpileup* (SAMtools utilities version 1.1, Li *et al*. 2009). A custom Python‐script screened the reads in the reference pool mapped to this position for presence of the alternative allele in the MA‐line individual and, if successful, removed it from the candidate list. All surviving candidate positions were manually curated by visualizing each candidate position along with the reference pool in IGV (v2.3.68, Robinson *et al*. 2011). This was necessary, because candidate positions contained several false positives due to paralog mismapping (Keightley *et al*. 2014a), PCR artefacts escaping duplicate removal and wrongly emitted variant calls.

The described approach has been shown to yield negligible false negatives (Keightley *et al*. 2014a). We tried to obtain independent confirmation for 36 candidate mutations (71%) by checking for their presence in the respective MA‐line by designing primers for the mutated region and Sanger‐sequencing the resulting PCR products for ten full‐siblings of the deep sequenced individual. Mutations occurred up to F_3_ should show in this sample at least once with a probability of 0.9989. Assuming that probability of occurrence is equal in all generations, we expected to confirm only about 80% of all assayed candidate positions because those occurring in the germline of the F_4_ generation will occur only in a single individual in F_5_ (Table S2).

Sites where a mutation could in principal be called according to our criteria were calculated separately for each individual. The mutation rate was directly calculated as ratio of mutations per number of callable sites over five generation, per haploid genome. Assuming a Poisson distribution, we used a Maximum Likelihood method as described in Haag‐Liautard *et al*. (2007) to estimate μ and the associated confidence intervals (*fitdistrplus* package, Delignette‐Muller and Dutang 2015). Mean number of mutations (separately for overall, SNM, deletion, and insertion rate) was used as lambda parameter for the Poisson distribution.

Using coalescence estimates of the population mutation parameter (θ) from 30 unlinked nongenic regions in European natural *C. riparius* populations (Oppold et al. 2017, Supplemental Text S1: Table 1) between 0.018 and 0.034, we estimate N_e_ for these populations following Charlesworth (2009): *N_e_ = θ/4μ*.

**Table 1:** Summary information on the number of callable sites, the total number of single base mutations, resulting mutation rate (μ) per generation and site, the number of single nucleotide mutations (SNMs) and the associated rate (μ SNM), number of alternative SNMs that occurred at the same site, number of insertions, deletions, transitions (Ts), and transversions (Tv) per mutation accumulation (MA) line

MA line	Number of callable sites	Number of mutations	μ	SNMs	μ(SNM)	Alternative SNM per site	Insertions	Deletions	Ts	Tv
A1	113,428,649	7	1.23 × 10^–8^	2	3.53 × 10^–9^	0	3	2	1	1
A2	126,279,134	4	6.34 × 10^–9^	2	3.17 × 10^–9^	1	1	1	1	0
A3	125,198,906	3	4.79 × 10^–9^	1	1.60 × 10^–9^	0	0	2	1	0
A4	119,583,116	7	1.17 × 10^–8^	4	6.69 × 10^–9^	0	2	1	2	2
A5	100,483,488	9	1.79 × 10^–8^	4	7.96 × 10^–9^	0	2	3	0	4
A6	129,246,425	4	6.19 × 10^–9^	2	3.09 × 10^–9^	0	0	2	0	2
A7	127,455,137	3	4.71 × 10^–9^	1	1.57 × 10^–9^	1	0	2	0	0
A8	128,191,107	7	1.09 × 10^–8^	4	6.24 × 10^–9^	0	0	3	2	2
A9	130,004,076	3	4.62 × 10^–9^	2	3.08 × 10^–9^	0	0	1	2	0
A10	129,317,412	4	6.19 × 10^–9^	4	6.19 × 10^–9^	0	0	0	4	0
SUM	1,229,187,450	51		26		2	8	17	13	11

We observed both TV and TS at sites with two alternative SNMs and these were therefore not listed to either of the two groups.

We investigated the mutational spectrum of *C. riparius*, by exploring the effect of base composition bias on the expected abundance, length, and nucleotide bias of monomer runs and CpG motives and the potential impact on occurrences of mutations (refer to Supplemental Text 2 for a detailed description of analyses). We then compared the expected mutational spectra of *C. riparius* and *D. melanogaster* (genome version 6.12 downloaded from http://flybase.org on 13/01/2017).

## Results

The mean sequence coverage ranged from 23.8x to 30.6x for the ten MA‐line individuals and was 69.9x for the reference pool, representing the parents (Table S1). The number of callable sites for the MA line individuals ranged between 113 and 130 Mb, covering up to 85% of the high complex regions of the genome. Overall, we identified 51 mutations (range 4–11 per individual) of which 26 were SPMs (range 2–6, Table 1). We attempted to validate 36 of these mutations (20 SNPs and 16 indels) by Sanger sequencing. We could confirm 31 of these (19 SNPs, 12 indels, Table S1). By experimental design (Fig. S1), we could principally not confirm mutations arisen in the last generation, that is 7.2 of the investigated 36 mutations assuming an equal μ across generations (Table S2). The number of six unconfirmed mutations was therefore within expectations as were the proportion of confirmed SNPs and indels and thus all 51 mutations were used for the calculation of μ.

Six mutations were found to be homozygous at least in some of the individuals that were Sanger‐sequenced for confirmation. This is not significantly different from the expected mean as inferred from a simulation (χ² = 0.343, *P* = 0.56, Table S2). One mutation was in an intron of an annotated gene. This is less than expected from the extent of the gene space (15%) in the draft genome.

Fifteen SNMs were transitions (Ts) and eleven transversions (Tv), resulting in a Ts/Tv ratio of 1.36. There were twice as many G/C to A/T mutations than vice versa (14 to 7), but this difference was not significant (χ² = 2.333, *P* = 0.13). We observed eight insertions and 17 deletions, a difference that only marginally deviated from random expectations (χ² = 3.240, *P* = 0.07). With few exceptions, indel mutations were associated with an A or T monomer stretch of at least five positions length (Table S1). Two Sanger confirmed mutations showed different bases in different individuals (A2 scaffold 31: 1191434, G>C and G>A and A7 185:145141, G>T and G>A, Table 1).

**Figure 1:**
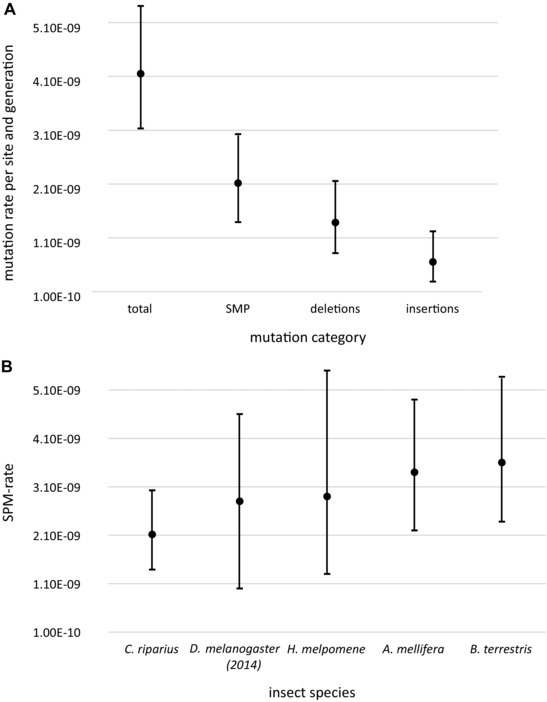
(A) Mean and confidence interval estimates for the overall single base mutation rate, the single nucleotide mutation (SNM) rate, the deletion, and insertion rate. (B) Comparison of the *C. riparius* SNM rate with 95% confidence intervals to rates of other insects (respectively Keightley *et al*. 2014a, b; Yang *et al*. 2015; Liu *et al*. 2016).

Our estimate of the haploid SNM rate was μ = 2.12 × 10^−9^ (95% CI: 1.39 × 10^−9^ – 3.03 × 10^−9^, Fig. 1A). We estimate N_e_ for European wild *C. riparius* populations to range between 2.16 × 10^6^ and 3.95 × 10^6^. The rate for single base deletions was μ_del_ = 1.39 × 10^−9^ (95% CI = 0.82 × 10^−9^ ‐ 2.07 × 10^−9^) and for insertions μ_ins_ = 0.65 × 10^−9^ (95% CI = 2.85 × 10^−10^ – 1.22 × 10^−9^). The overall mutation rate for all single base mutations was thus 4.15 × 10^−9^ (95% CI = 3.13 × 10^−9^ ‐ 0.54 × 10^‐8^, Fig. 1A).

The mutational spectrum of *C. riparius* was found to be strongly shifted from SNM to indel mutations, which preferentially occurred in A/T monomer nucleotide runs (21 out of 25, Table S1). Monomer nucleotide runs in the *C. riparius* draft genome have a strong A/T bias (98%, Supplemental Text 2) and are product of and contribute over proportionally to the overall high A/T content (69%) of the genome (Oppold et al. 2017). The comparison of the abundance and length of monomer nucleotide runs in the genomes of *C. riparius* and *D. melanogaster* revealed a larger number of longer monomer nucleotide runs in *C. riparius* (Supplemental Text 2: Fig. 5), whereas *D. melanogaster* has a larger number of CpG dinucleotide runs (Supplemental Text 2: Fig. 6).

## Discussion

We here present a direct estimate of the spontaneous mutation rate in *C. riparius*, a valuable resource from a nonmodel dipteran as additional representative for the vast biodiversity of insects. We were able to confirm the expected proportion of mutation candidates by Sanger sequencing (83.3%), suggesting a very low false‐positive rate that is comparable to previous studies (Keightley *et al*. 2014a). Together with the known low false‐negative rate of the applied bioinformatics pipeline (Keightley *et al*. 2014a) the presented values likely are accurate estimates. The estimated SNM rate reported here is in the range, although at the lower margin, of estimates from both MA‐lines and single generation parent‐offspring approaches in *D. melanogaster* (2.8 × 10^−9^ to 5.49 × 10^−9^, Keightley *et al*. 2009; Schrider *et al*. 2013; Keightley *et al*. 2014a) or *H. melpomene* (2.9 × 10^−9^, Keightley *et al*. 2015), with broadly overlapping confidence intervals. It is however, much lower than the recently reported rates for *A. mellifera* and *B. terrestris* (Yang *et al*. 2015, Liu *et al*. 2016, Fig. 1B). Due overlapping confidence interval of μ among insects, there is an ongoing debate, whether mutation rates are rather constant in insects (Liu *et al*. 2016). Insect sociality was shown to be associated with increased recombination rates (Wilfert *et al*. 2007) what might also affect their mutation rates, even though this could so far not be proven (Liu *et al*. 2016). The lower μ of *C. riparius* might provide further input for the resolution of possibly different μ among insects.

The estimate of the *C. riparius* effective population size is comparable to but slightly higher than both *D. melanogaster* and *H. melpomene* (∼1.4 × 10^6^ or ∼2 × 10^6^ for *D. melanogaster* and ∼2 × 10^6^ for *H. melpomene* (Keightley *et al*. 2014a; Keightley *et al*. 2015). The effective population size of *A. mellifera*, however, is estimated to be a magnitude lower (Wallberg *et al*. 2014). The emerging relation of increasing μ with decreasing N_e_ among these four insect species (comparable N_e_ data for *B. terrestris* is not available, *cf*. Lattorf *et al*. 2016) may be taken as support for the drift‐barrier hypothesis (Lynch *et al*. 2016), stating that the realised μ of a species is determined by the balance between selection and drift and thus N_e_ and mutation rates should be negatively correlated.

Even though not significantly different, the observed bias toward G/C > A/T mutations is in line with the high A/T content of the *C. riparius* genome (G/C content 31%, Oppold *et al*. 2017). This A/T bias of the *C. riparius* genome is probably also responsible for the observed shift from SNMs to indel mutations compared to other organisms (Supplemental Text 2). It leads statistically to an over proportional increase in AT monomer runs and thus increases the probability for mutations to occur at such sites. Indeed, indel mutations were significantly more often observed in monomer stretches than expected, and their mutation rate increased exponentially with stretch length. At the same time, the high A/T content statistically decreases the amount of CpG sites, known to be target sites for C>T transitions (Supplemental Text 2). Comparison to *D. melanogaster* (G/C content 42%) suggested that genomic base composition is a driver of the mutational spectrum. The more biased the genome base composition towards A/T, the more indel mutations and the less nucleotide substitutions are expected (Supplement Text 2).

The phenomenon of two different mutations at the same site in different individuals of the same MA line (Table 1) was recently reported also in *D. melanogaster* (Keightley *et al*. 2014a) and plausibly explained by a premeiotic mutation cluster with a subsequent error‐prone mismatch repair late in the development of the germ line in the immediately previous generation (Goodman 2002). Our results indicate that this creation of threefold degenerate sites may be a relatively frequent process, meriting future attention.

The here presented experimental set‐up combines short‐term mutation accumulation lines with the information of the parental genotypes and is thus an efficient approach to estimate μ in many organisms even without high quality reference genomes. While identifying a substantial number of mutations, the effort in terms of time (five generations of about 28 days each) and deep sequencing of ten individuals and the reference pool appeared reasonable. Yet, over such a short period, all mutations can still occur in heterozygous fashion, thus revealing the full mutational spectrum (Table S2). The applied experimental design is furthermore promising to determine the influence of demographic and environmental factors and/or anthropogenic substances on the evolutionary relevant germline mutation rate.

## Supporting information


**Figure S1**. Scheme of the experimental design.
**Table S1**. List of identified mutations. Given are the mutation accumulation line (Ma) in which theiy were identified, the scaffold and base pair position, the mutation type, the sequence context 10 bp up‐ and downstream, the base in the reference pool, the mutated base, whether the mutation is a transistion (TS) or transversion (TV), mutation from A/T to G/C or vice versa, indication whether mutation confirmation was attempted via Sanger sequencing and whether the mutation could be confirmed and if so, if it occurred in heterozygous (hetero) and/or homozygous (homo) state.
**Table S2**. Derivation of probabilities to observe a mutation in either heterozygous or homozygous state, depending on the generation of their occurrence.
**Table 1**: Estimated population mutation parameter θ and effective population size for each of five natural *C. riparius* populations (refer to Oppold *et al*. 2017 for detailed sample information).
**Figure 1**. Influence of base composition bias on the expected abundance and length of monomer and CpG runs. Left) Logarithmic plot of mean expected monomer runs per Mb as a function of their length for different base compositions. Right) Logarithmic plot of mean expected CpG runs per Mb as a function of their length for different base compositions.
**Figure 2**. Left) Logarithmic plot of observed monomer runs frequencies for all (red), A/T (blue) and G/C (green) runs. Right) Expected ratio of A/T to G/C monomer runs as a function of the AT content.
**Figure 3**. Logarithmic plot of expected (dashed lines, 95% confidence interval) and observed number of monomer runs per Mb (solid line) for left) A/T runs and right) G/C runs.
**Table 1**. Basic statistics of the monomer run content and CpG positions of the high complexity regions of the *C. riparius* reference genome.
**Figure 4**. Estimated mutation rates for different monomer run lengths per run site and generation. Please note the logarithmic scale.
**Figure 5**. Comparison of genomic monomer runs content among *C. riparius* and *D. melanogaster*.
**Figure 6**. Comparison of genomic CpG runs content among *C. riparius* and *D. melanogaster*.
