# Supplementary material for: Direct estimation of the spontaneous mutation rate by short‐term mutation accumulation lines in Chironomus riparius
Source: Evol Lett. 2017 May 11;1(2):86–92. doi: 10.1002/evl3.8 (PMC6121839; doi:10.1002/evl3.8)
Supplement: Supplementary file 1 — Figure S1. Scheme of the experimental design. Table S1. List of identified mutations. Given are the mutation accumulation line (Ma) in which theiy were identified, the scaffold and base pair position, the mutation type, the sequence context 10 bp up‐ and downstream, the base in the reference pool, the mutated base, whether the mutation is a transistion (TS) or transversion (TV), mutation from A/T to G/C or vice versa, indication whether mutation confirmation was attempted via Sanger sequencing and whether the mutation could be confirmed and if so, if it occurred in heterozygous (hetero) and/or homozygous (homo) state. Table S2. Derivation of probabilities to observe a mutation in either heterozygous or homozygous state, depending on the generation of their occurrence. Table 1: Estimated population mutation parameter θ and effective population size for each of five natural C. riparius populations (refer to Oppold et al. 2017 for detailed sample information). Figure 1. Influence of base composition bias on the expected abundance and length of monomer and CpG runs. Left) Logarithmic plot of mean expected monomer runs per Mb as a function of their length for different base compositions. Right) Logarithmic plot of mean expected CpG runs per Mb as a function of their length for different base compositions. Figure 2. Left) Logarithmic plot of observed monomer runs frequencies for all (red), A/T (blue) and G/C (green) runs. Right) Expected ratio of A/T to G/C monomer runs as a function of the AT content. Figure 3. Logarithmic plot of expected (dashed lines, 95% confidence interval) and observed number of monomer runs per Mb (solid line) for left) A/T runs and right) G/C runs. Table 1. Basic statistics of the monomer run content and CpG positions of the high complexity regions of the C. riparius reference genome. Figure 4. Estimated mutation rates for different monomer run lengths per run site and generation. Please note the logarithmic scale. Figure 5. Comparison of genomic [file EVL3-1-86-s001.docx]

**Supplementary Info**

**Estimation of the spontaneous mutation rate by short term mutation accumulation lines in the non-biting midge *Chironomus riparius***

Ann-Marie Oppold & Markus Pfenninger

Figure S1. Scheme of the experimental design.


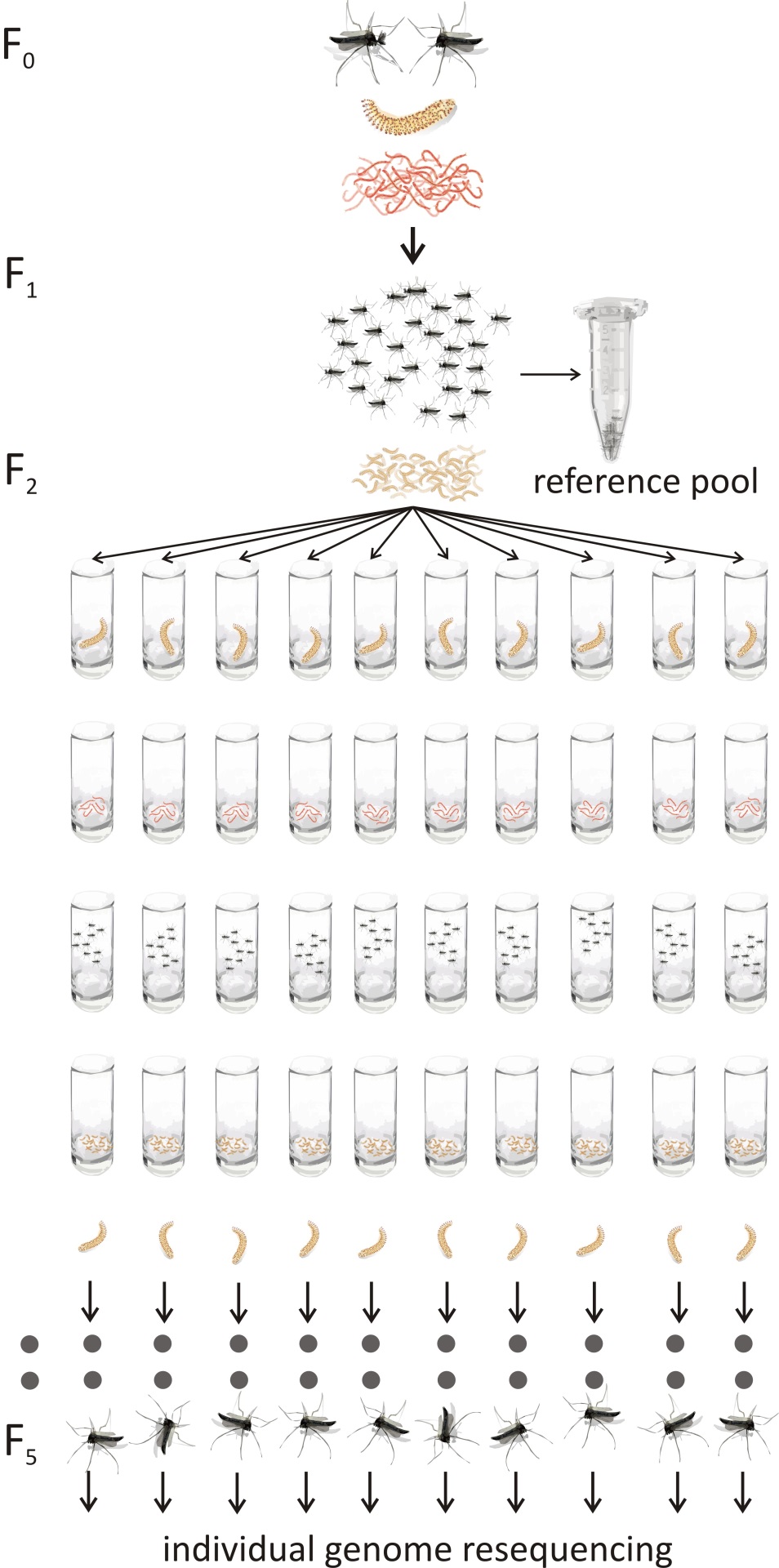


Table S1. List of identified mutations. Given are the mutation accumulation line (Ma) in which theiy were identified, the scaffold and base pair position, the mutation type, the sequence context 10 bp up- and downstream, the base in the reference pool, the mutated base, whether the mutation is a transistion (TS) or transversion (TV), mutation from A/T to G/C or vice versa, indication whether mutation confirmation was attempted via Sanger sequencing and whether the mutation could be confirmed and if so, if it occurred in heterozygous (hetero) and/or homozygous (homo) state.

| Ma line | scaffold | position | mutation type | context | reference base | mutation | TS/TV | gene | A/T> C/G | C/G> A/T | Sanger checked | confirmed | allelic state |
| --- | --- | --- | --- | --- | --- | --- | --- | --- | --- | --- | --- | --- | --- |
| A1 | scaffold1 | 1408155 | insertion | ATTTATAGTA**T**TTTTTAACTT | A | AT |  |  |  |  | yes | yes | hetero |
|  | scaffold31 | 219294 | SNP | TTTTACATCC**C**AGACGAATTT | G | C | TV |  |  |  | yes | yes | homo |
|  | scaffold45 | 344749 | SNP | GCTTGTGGTT**A**ACACAGTCAG | G | A | TS |  |  | + | yes | yes | homo/hetero |
|  | scaffold150 | 125441 | insertion | TTGATTTTTG**A**AAAAAAGCGA | G | GA |  |  |  |  | yes | yes | hetero |
|  | scaffold200 | 81719 | insertion | AATGATAAAG**A**AATGTATCAA | G | GA |  |  |  |  | yes | no | hetero |
|  | scaffold270 | 8038 | deletion | TAAGTTCCTA**-**TTTTTTTTTA | AT | A |  | yes |  |  | yes | yes | hetero |
|  | scaffold283 | 32972 | deletion | TCAATTCACC**-**AAAAAAATGA | CA | C |  |  |  |  |  |  |  |
| A2 | scaffold25 | 413316 | SNP | CTCAGAATTG**T**ATAGATGATG | C | T | TS |  |  | + | yes | yes | hetero |
|  | scaffold31 | 1191434 | SNP | GAAAAAAAGA**A**ATTCAAATAG | G | A | TS |  |  | + | yes | yes | hetero |
|  | scaffold69 | 447444 | insertion | CCAAATCATG**T**TTTTTTTTTG | G | GT |  |  |  |  | yes | yes | hetero |
|  | scaffold412 | 200133 | deletion | GCAGCATACC**-**AAAAAAAATC | CA | C |  |  |  |  | yes | yes | hetero |
| A3 | scaffold7 | 509054 | deletion | ACAAAATCCA**-**TTTTTTTTTA | AT | A |  |  |  |  | yes | yes | hetero |
|  | scaffold40 | 219059 | deletion | CGTTGATTGC**-**AAAAAAAATG | CA | C |  |  |  |  | yes | yes | hetero |
|  | scaffold623 | 9623 | SNP | TTCGAAAGAG**A**ACAAATTAAA | G | A | TS |  |  | + |  |  |  |
| A4 | scaffold5 | 386370 | insertion | AATTTATAAC**A**AAAAAAATTA | C | CA |  |  |  |  | yes | no | hetero |
|  | scaffold26 | 201140 | insertion | TACAAGAAAC**A**AAAAAAAAAA | C | CA |  |  |  |  | yes | yes | homo |
|  | scaffold50 | 791827 | SNP | TAGTCGTAAG**T**TAGAAAATTA | G | T | TV |  |  | + | yes | yes | hetero |
|  | scaffold154 | 181197 | SNP | TACAAATATT**T**ACTCACGAAG | G | T | TV |  |  | + | yes | yes | hetero |
|  | scaffold163 | 312123 | SNP | ATTATTACGG**C**CTCCATGCAA | T | C | TS |  | + |  | yes | yes | hetero |
|  | scaffold167 | 76594 | deletion | CAGTTTTTTC**-**AAAAAAAATT | CA | C |  |  |  |  | yes | yes | hetero |
|  | scaffold985 | 8371 | SNP | ACAAATTCAC**G**TGGCTCCAGG | A | G | TS |  | + |  |  |  |  |
| A5 | scaffold17 | 651170 | SNP | TAAAGGCAAA**C**CCAAAAAAAA | A | C | TV |  | + |  | yes | yes | homo/hetero |
|  | scaffold29 | 336162 | insertion | TGTCAAAACA**T**TTTTTTTTTT | A | AT |  |  |  |  |  |  |  |
|  | scaffold59 | 149577 | SNP | TAGGGTTGAT**T**CCATCAAATT | A | T | TV |  |  |  |  |  |  |
|  | scaffold121 | 54814 | deletion | ATTATTTAAT**-**GATGTTACGC | TG | T |  |  |  |  | yes | no | hetero |
|  | scaffold189 | 137440 | deletion | ACAAGATCAA**-**TTTACATACG | AT | A |  |  |  |  |  |  |  |
|  | scaffold195 | 134136 | insertion | TCCATAAAAG**C**CCCCCCCCCC | G | GC |  |  |  |  |  |  |  |
|  | scaffold267 | 22672 | SNP | GTAAGTCTGT**A**CATTCTTCTC | C | A | TV |  |  | + | yes | yes | homo |
|  | scaffold276 | 23996 | SNP | GAGATCTGGA**C**CTACTCTAAG | G | C | TV |  |  |  | yes | yes | homo/hetero |
|  | scaffold326 | 93251 | deletion | GAATCATTCG**-**CCAAACCTTG | GC | G |  |  |  |  |  |  |  |
| A6 | scaffold243 | 29748 | SNP | CTCTAGGTCC**T**TTCCATTAAA | G | T | TV |  |  | + | yes | yes | hetero |
|  | scaffold256 | 114637 | SNP | ATTCCATTCG**T**GTTGAGGAAT | A | T | TV |  |  |  | yes | yes | hetero |
|  | scaffold382 | 67246 | deletion | CTGTAAATAA**-**TTTTTTTTTG | AT | A |  |  |  |  | yes | no | hetero |
|  | scaffold581 | 46883 | deletion | TAACTTAAGA**-**TTTTTTTTTT | AT | A |  |  |  |  | yes | yes | hetero |
| A7 | scaffold185 | 145141 | SNP | AAAACAGCCG**A**GAACTAGCGG | G | A | TS |  |  | + |  |  |  |
|  | scaffold282 | 7409 | deletion | TTTCAAGAAC**-**TTTTTTTGCA | CT | C |  |  |  |  | yes | yes | hetero |
|  | scaffold637 | 12063 | deletion | GGGTTTACCA**-**TTTTTTTTGG | AT | A |  |  |  |  | yes | yes | hetero |
| A8 | scaffold5 | 658683 | deletion | GCATTTTGAC**-**AAAAAAAAAT | CA | C |  |  |  |  |  |  |  |
|  | scaffold26 | 630176 | SNP | ATTACAAAAA**A**AAATTCGCAG | T | A | TV |  |  |  | yes | no | hetero |
|  | scaffold49 | 749494 | SNP | ATTACAAAAA**A**AAATTCGCAG | A | C | TV |  | + |  | yes | yes | hetero |
|  | scaffold75 | 243457 | deletion | ACTTCCATCA**-**TTTTTTTTTG | AT | A |  |  |  |  | yes | yes | hetero |
|  | scaffold189 | 289120 | SNP | CAATTGTTGA**C**GGATCTTATA | T | C | TS |  | + |  | yes | no | hetero |
|  | scaffold454 | 67203 | deletion | AGAAAAAAAG**-**TTTTTTTTGT | GT | G |  |  |  |  | yes | yes | hetero |
|  | scaffold721 | 3700 | SNP | GATCCATCAA**T**ATTGTCTGTT | C | T | TS |  |  | + |  |  |  |
| A9 | scaffold935 | 4628 | deletion | TAATAGTTTG**-**AAAAAAAAAT | GA | G |  |  |  |  |  |  |  |
|  | scaffold1577 | 4603 | SNP | GTATTTTTCA**G**CAGTTCAAAA | A | G | TS |  | + |  | yes | yes | hetero |
|  | scaffold1577 | 4957 | SNP | CATCTATCCA**T**TCTGTCATTA | C | T | TS |  |  | + |  |  |  |
| A10 | scaffold8 | 773458 | SNP | AACAACAGCT**A**AGTCAATGCA | G | A | TS |  |  | + | yes | yes | homo/hetero |
|  | scaffold32 | 504864 | SNP | CGTGTCTGTA**G**GGACGTGTCT | A | G | TS |  | + |  | yes | yes | homo/hetero |
|  | scaffold146 | 343308 | SNP | ATGTAACACA**T**TGTACAGTTA | C | T | TS |  |  | + | yes | yes | hetero |
|  | scaffold304 | 122155 | SNP | ATCAATAGCT**A**CACATCAGCT | C | T | TS |  |  | + |  |  |  |

Table S2. Derivation of probabilities to observe a mutation in either heterozygous or homozygous state, depending on the generation of their occurrence.

**Supplemental Text 1**

**Estimation of the effective population size of natural *C. riparius* populations**

Effective population sizes N_e_ were calculated from five different *C. riparius* populations, making use of Pool-Seq data (Oppold *et al.* 2017, European Nucleotide Archive study accession number PRJEB19848). To obtain haplotype information for multiple loci from Pool-Seq data, we used the individual read information of the data ([Pfenninger *et al.* 2015](#_ENREF_10)) and extracted 30 loci of 150bp length, containing at least five SNPs, from the PoPoolation2 F_ST_ output file. For all loci, we considered 15 reads per population, thus representing haplotypic information of 15 chromosomes. We used Migrate-n (v3.6.5, [Beerli 2006](#_ENREF_2); [Beerli & Felsenstein 2001](#_ENREF_3)) for Bayesian inference of the population mutation parameter (θ). Parameters were set as described by Pfenninger *et al.* ([2015](#_ENREF_10)). Estimated θ ranged between 0.018 and 0.034. Accordingly, we calculated the expected N_e_ following Charlesworth ([2009](#_ENREF_5)): N_e_ = θ/4µ.

**Table 1**: Estimated population mutation parameter θ and effective population size for each of five natural *C. riparius* populations (refer to Oppold *et al.* 2017 for detailed sample information).

| **population ID** | **θ** | **N_e_** |
| --- | --- | --- |
| **MF** | 0.029 | 3.45E+06 |
| **MG** | 0.030 | 3.57E+06 |
| **NMF** | 0.034 | 3.95E+06 |
| **SI** | 0.028 | 3.30E+06 |
| **SS** | 0.018 | 2.15E+06 |

**Supplemental Text 2**

**Influence of genomic base composition on mutation targets in *C. riparius***

Even though the overall single base mutation rate of *C. riparius* was very similar to those published for other insects (e.g. ([Keightley *et al.* 2014](#_ENREF_6); [Keightley *et al.* 2009](#_ENREF_7)), the mutational spectrum found here was strongly shifted from SNM to indel mutations. The latter occurred preferentially in A/T monomer nucleotide runs (21 out of 25). After Sanger sequencing confirmation that this is not due to mapping or assembly artefacts, the bias raised the suspicion that perhaps the high genomic AT content could be responsible for this pattern, moreover since such a bias could also affect the abundance of CpG motives, known for their susceptibility to point mutations ([Bird 1980](#_ENREF_4)).

To infer potential causes for this shift in the mutational spectrum in *C. riparius*, we first explored the effect of base composition bias on the expected abundance, length and nucleotide bias of monomer runs and CpG motives. We simulated 1000 DNA stretches of 1 Mb length by randomly drawing bases from different base compositions (50%, 60%, 70% and 80% AT content). We then recorded the frequency length distribution of resulting monomer stretches longer than 5 bp and CpG motives of all length.

**Figure 1.** Influence of base composition bias on the expected abundance and length of monomer and CpG runs. Left) Logarithmic plot of mean expected monomer runs per Mb as a function of their length for different base compositions. Right) Logarithmic plot of mean expected CpG runs per Mb as a function of their length for different base compositions.

Both the expected mean number and mean length of all monomer runs increased with increasing base composition bias (Figure 1, left), comprising between 1.6% of all base positions for 50% AT content and 6.9% for 80% AT content. The ratio of A/T vs. G/C monomer runs increased exponentially from 1 (50% AT content) to 680 (80% AT content, Figure 1, right). As expected, the opposite was true for CpG motives that were more abundant and longer with lower AT content (Figure 1, right), comprising between 3.1 % (80% AT content) and 14.7% (50% AT content) of the positions. This showed that just for statistic reasons, the abundance, length distribution and bias in monomer and CpG runs depends strongly on the genomic base composition of the respective organism.

We then counted the monomer runs between 5-24 bp in the reference genome of *C. riparius*. In total, there were more than 1.8 million of such monomer runs. The length distribution of the monomer runs was roughly exponentially declining (Figure 2).

**Figure 2.** Left) Logarithmic plot of observed monomer runs frequencies for all (red), A/T (blue) and G/C (green) runs. Right) Expected ratio of A/T to G/C monomer runs as a function of the AT content.

The vast majority of them were A/T runs (1.82 million) versus only 43778 G/C runs (Table 1, ratio 41.6). This makes up 5.87% and 0.14% of the reference genome draft and its high complexity regions, respectively. In terms of base pair composition, the A/T content of the monomer runs (98%) exceeded by far the genome-wide average (69%, ([Oppold *et al.* 2016](#_ENREF_9))) and is thus contributing to the high A/T content of the *C. riparius* genome.

To infer whether monomer stretches are just random products of the general base pair composition of *C. riparius*, we simulated 1000 DNA stretches of 1 Mb each by randomly drawing from the observed base pair distribution in the genome (A 34.5%, T 34.5%, G 15.5%, C 15.5%) and calculated the 95% confidence interval for the occurrence of monomer runs from 5 – 24 base pairs. Normalising the observed number of monomer runs to 1 Mb for comparison showed that for both A/T and G/C runs, the observed number of monomer runs was greater than the expected. The difference was lower for shorter runs and increased for longer runs (Figure 3).

**Figure 3.** Logarithmic plot of expected (dashed lines, 95% confidence interval) and observed number of monomer runs per Mb (solid line) for left) A/T runs and right) G/C runs.

This suggested that not only random processes are responsible for the observed abundance and length distribution of monomer runs in the *C. riparius* genome. This interpretation is supported by the fact that indel mutations occurred significantly more often in monomer stretches longer than 5 bp than in the rest of the genome (21 versus 4 in 5.87% vs. 94.13% of the genome, respectively, Χ² = 345.1 p < 0.0001). In addition, the probability for an indel mutation (*i.e.* the mutation rate) strongly increased with the length of the stretch (Figure 4 ([Bacon *et al.* 2001](#_ENREF_1))).

**Table 1.** Basic statistics of the monomer run content and CpG positions of the high complexity regions of the *C. riparius* reference genome.

|  | Total number | Base pairs | Proportion | Percent of the genome |
| --- | --- | --- | --- | --- |
| A/T | 1,824,305 | 10,606,267 | 0.977 | 5.87 |
| G/C | 43,778 | 245,544 | 0.023 | 0.14 |
| All | 1,868,083 | 10,851,811 |  | 6.01 |
| CpG | 4,259,457 | 9,828,478 |  | 5.44 |

Even though there were more deletions than insertions observed in monomer runs (14 : 7), this difference was not significant, based on a 1:1 expectation (Χ² = 2.33 p = 0.127). There was also no significant trend for insertions or deletions to occur preferentially in either short (< 9) or long (>= 9) monomer runs (Fisher’s exact test p = 0.280). There was no significant difference in mutability of A/T versus G/C runs, taking their different abundance in the genome into account (21 and 1 mutations observed, expected frequencies 0.987 and 0.023, respectively, Χ² = 0.562 p = 0.453). However, despite the comparatively large number of mutations available for analysis in this study, these results may change with an increasing number of observed mutations.

**Figure 4.** Estimated mutation rates for different monomer run lengths per run site and generation. Please note the logarithmic scale.

These findings suggested the following model: Short monomer runs occur by chance, respectively are an universal unavoidable effect of the limited number of different DNA bases. Above a certain length threshold, their further dynamic is driven by indel mutations to which these monomer runs are inherently increasingly susceptible with increasing length ([Lai & Sun 2003](#_ENREF_8)). The more the genomic base composition deviates from uniformity, the more and the longer monomer runs the respective genome has and thus more indel mutations are expected. In addition, in case of an AT bias as observed here, the number of CpG sites decreases and thus the probability for point mutations which should shift the ratio even further in favour of indel mutations.

To test this prediction with empirical data, we compared the monomer and CpG run content among *C. riparius* and *D. melanogaster* (genome version 6.12 downloaded from flybase.org on 13.1.2017). The base pair composition of *D. melanogaster* was estimated from the data to 57.6% AT and 42.4% GC as opposed to 69.0% AT and 31.0% GC in *C. riparius*. In both species, the observed monomer run abundance and length was larger than expected from the base composition (Figure 5, left). However, apart from monomers of 9-11 bp length, there were consistently more repeats per Mb in *C. riparius*, which was mirrored in the much higher total abundance (Figure 5, right) and proportion of the genome (6.01% vs. 4.08%, respectively). Therefore, there are much more opportunities for indel mutations in monomer runs in the *C. riparius* genome compared to *D. melanogaster*. It would have been interesting to compare the indel mutation rates for different monomer run length classes, however, there is not enough data available for *D. melanogaster*.

**Figure 5.** Comparison of genomic monomer runs content among *C. riparius* and *D. melanogaster*.

Left: Logarithmic plot of the observed (solid line) log number of monomer runs per 1 Mb as a function of their length with the 95% confidence interval as expected according to the genomic base pair composition. Right: Mean number of monomer runs larger 5 bp per 1 Mb.

**Figure 6.** Comparison of genomic CpG runs content among *C. riparius* and *D. melanogaster*. left) Logarithmic plot of the observed (solid line) log number of CpG runs per 1 Mb as a function of their length with the 95% confidence interval as expected according to the genomic base pair composition. right) Mean number of CpG runs per 1 Mb.

In contrast, the content of CpG runs was mostly within the statistical expectations, except for the longest (and rarest) runs (Figure 6, left). In contrast to monomer runs, the picture was reversed, *D. melanogaster* harboured more CpG motives per Mb than *C. riparius*, according to the expectations from the respective genomic AT content (Figure 6, right). Both findings could explain the observed shift in mutational spectrum among the two species.

In conclusion, the observed accumulation of indel mutations in A/T mononucleotide runs and the relatively low ratio of SMP to indels in *C. riparius* is most probably due to the AT bias of the entire genome which increases both the relative and absolute number and length of potential indel mutation targets and simultaneously decreases the respective potentially particular susceptive CpG point mutation positions.

**References**

Bacon AL, Dunlop MG, Farrington SM (2001) Hypermutability at a poly (A/T) tract in the human germline. *Nucleic Acids Research* **29**, 4405-4413.

Beerli P (2006) Comparison of Bayesian and maximum-likelihood inference of population genetic parameters. *Bioinformatics* **22**, 341-345.

Beerli P, Felsenstein J (2001) Maximum likelihood estimation of a migration matrix and effective population sizes in n subpopulations by using a coalescent approach. *Proceedings of the National Academy of Sciences of the United States of America* **98**, 4563-4568.

Bird AP (1980) DNA methylation and the frequency of CpG in animal DNA. *Nucleic Acids Research* **8**, 1499-1504.

Charlesworth B (2009) Effective population size and patterns of molecular evolution and variation. *Nature Reviews Genetics* **10**, 195-205.

Keightley PD, Ness RW, Halligan DL, Haddrill PR (2014) Estimation of the Spontaneous Mutation Rate per Nucleotide Site in a *Drosophila melanogaster* Full-Sib Family. *Genetics* **196**, 313-320.

Keightley PD, Trivedi U, Thomson M*, et al.* (2009) Analysis of the genome sequences of three Drosophila melanogaster spontaneous mutation accumulation lines. *Genome Research*, gr. 091231.091109.

Lai Y, Sun F (2003) The relationship between microsatellite slippage mutation rate and the number of repeat units. *Molecular Biology and Evolution* **20**, 2123-2131.

Oppold A-M, Schmidt H, Rose M*, et al.* (2016) *Chironomus riparius* (Diptera) genome sequencing reveals the impact of minisatellite transposable elements on population divergence. *bioRxiv*, 080721.

Pfenninger M, Patel S, Arias-Rodriguez L*, et al.* (2015) Unique evolutionary trajectories in repeated adaptation to hydrogen sulphide-toxic habitats of a neotropical fish (*Poecilia mexicana*). *Molecular Ecology* **24**, 5446-5459.
